# Discovery of a phylogenetically distinct poxvirus in diseased *Crocodilurus amazonicus* (family Teiidae)

**DOI:** 10.1007/s00705-021-04975-6

**Published:** 2021-02-12

**Authors:** Kerstin Seitz, Anna Kübber-Heiss, Angelika Auer, Nora Dinhopl, Annika Posautz, Marlene Mötz, Alexandra Kiesler, Claudia Hochleithner, Manfred Hochleithner, Gregor Springler, Annika Lehmbecker, Herbert Weissenböck, Till Rümenapf, Christiane Riedel

**Affiliations:** 1 Department of Pathobiology, Institute of Virology, University of Veterinary Medicine, Veterinärplatz 1, 1210 Vienna, Austria; 2 Department of Interdisciplinary Life Sciences, Research Institute of Wildlife Ecology, University of Veterinary Medicine, Vienna, Austria; 3 Department of Pathobiology, Institute of Pathology, University of Veterinary Medicine, Vienna, Austria; 4 Tierklinik Strebersdorf, Vienna, Austria; 5 Labor In Vitro GmbH, Vienna, Austria; 6 IDEXX Vet Med Labor GmbH, Kornwestheim, Germany

## Abstract

A novel poxvirus was discovered in *Crocodilurus amazonicus* (Teiidae) presenting with a debilitating skin disease. The generated first genome sequence of a reptilian poxvirus revealed the closest phylogenetic relationship to avipoxviruses, highlighting potential virus exchanges between avian and reptilian species.

**Supplementary Information:**

The online version contains supplementary material available at 10.1007/s00705-021-04975-6.

Poxviruses are large enveloped viruses containing a double-stranded DNA genome of 130-360 kb. They are important pathogens of high public health and economic impact and are able to infect a wide range of host species, ranging from insects to mammals. Within the family *Poxviridae*, two subfamilies (*Chordopoxvirinae* and *Entomopoxvirinae*) have been defined based on their hosts, which are either vertebrates or insects [1, 2]. The subfamily *Chordopoxvirinae* is divided into 18 genera, which include a total of 52 species [3].

Poxviruses infecting non-mammalian species belong to the genera *Avipoxvirus* and *Crocodylidpoxvirus*. As also observed in mammalian hosts, poxviruses of avian and crocodilian species are primarily associated with skin lesions but can also affect the upper respiratory and gastrointestinal tract [4, 5]. Poxvirus infections of reptiles other than crocodilians have been described in the literature, but no sequence information or further characterization of these viruses is available [6–11].

The species affected in this study, *Crocodilurus amazonicus* (“crocodile tegu”), is part of the family Teiidae. These lizards are native to the Amazon and Orinoco basins in South America [12] and belong to one of only two genera of living semi-aquatic teiids, and as such, they inhabit seasonally flooded forested areas near riverbanks or other watercourses. Their mean body temperature, 31.2°C, is higher than the environmental temperature but relatively low compared to that of other teiids, which could be due to their association with water habitats. Their variable diet consists of insects, other small reptiles, and frogs [13, 14].

In 2019, five *C. amazonicus*, owned by a private collector in Austria, were presented with skin lesions and weight loss. Due to the severity of the clinical signs, one animal had to be euthanized and was sent for further diagnostic evaluation to the pathologists of the Research Institute of Wildlife Ecology, University of Veterinary Medicine, Vienna, Austria. The animal was underweight and had multiple elevated, partially ulcerated skin lesions with a diameter of up to 4 mm on the back and the dorsal areas of head, neck, and tail that did not extend into the underlying muscle (Fig. 1A). Lesions were subsequently analyzed histologically, revealing multifocally and focally extensive epidermal hyperplasia of up to 10-15 times the normal thickness with a severely thickened stratum spinosum forming rete ridges. Keratinocytes showed ballooning degeneration and contained large eosinophilic, intracytoplasmic viral inclusion bodies (Bollinger bodies) up to 20 µm in size (Fig. 1B). Large numbers of poxvirus-like particles were detected within these lesions by transmission electron microscopy (Fig. 1C).

**Fig. 1:**
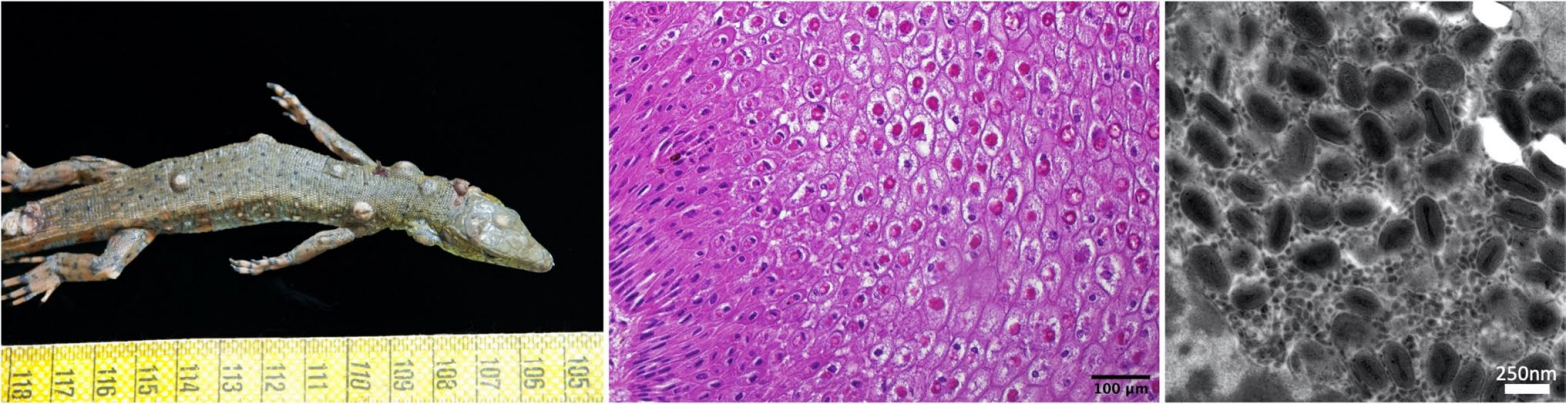
Pathologic and histologic examination. (A) Macroscopic presentation of skin lesions, represented by multiple, up to lentil-sized, elevated, partially ulcerated foci. This scale bar is in centimeters. (B) Hematoxylin-eosin-stained tissue section of an affected area of skin, displaying severe ballooning of the cells and prominent, eosinophilic intracytoplasmatic inclusion bodies. (C) Multiple poxvirus-like particles in skin lesions detected by transmission electron microscopy of uranyl-acetate-stained tissue sections.

A sample taken from a skin lesion tested positive for the presence of poxvirus DNA when a pan-poxvirus PCR for high-GC-content poxviruses developed by Li et al. was used [15]. Comparison of this 630-bp amplicon, which corresponded to part of the gene encoding the DNA-dependent RNA polymerase subunit rpo147 ortholog, to sequences available in public databases revealed the closest relationship, with 80% sequence identity, to members of the genus *Avipoxvirus*. This isolate was tentatively named "teiidaepox virus 1" (TePV-1). Cultivation in freshly isolated chicken embryo fibroblasts was attempted at different temperatures (22, 28 and 37°C), but this did not result in detectable virus replication, either by PCR testing of the supernatant or the development of a cytopathic effect. In order to characterize the virus further, we determined the sequence of the virus genome, employing a combination of Illumina sequencing technology (150-bp paired-end reads) and nanopore sequencing (MinION, Oxford Nanopore Technologies [ONT]) using DNA extracted from skin. Briefly, an aseptically dissected piece of skin containing lesions of approximately 10 mg was mechanically homogenized in 60 µl of PBS in a TissueLyser II at 30 Hz for 3 min (QIAGEN). For nanopore sequencing, 120 µl of lysis buffer was added, and the rest of the DNA preparation was performed following the manufacturer’s instructions for preparation of DNA from tissues using a QIAamp DNA Mini Kit. For Illumina sequencing, DNA was extracted using an NEB genomic DNA extraction kit according to the manufacturer’s instructions. The quality of the DNA preparation was checked using Genomic DNA ScreenTape (Agilent) on a 4200 TapeStation (Agilent) at the VetCore genomics facility. The library for Illumina sequencing was prepared using an NEBNext Ultra II DNA Library Prep Kit (New England Biolabs) according to the manufacturer’s protocol and quality controlled using a fragment analyzer at the Vienna BioCenter Core Facilities before being sequenced using Illumina MiSeq chemistry. The raw reads were quality controlled, and adapter sequences were removed before commencing data analysis. For nanopore sequencing, the DNA was processed according to the protocol for sequencing of genomic DNA by ligation (SQK-LSK109) provided by ONT, and the library was loaded onto a Nanpore Flongle Flowcell (ONT).

Before contig assembly, the nanopore reads were aligned to the genome sequence of penguinpox virus using bowtie2-2.2.8 [16]. Nanopore reads aligning to this sequence were subsequently used for contig assembly of the Illumina reads with SPAdes 3.14.0 [17]. This straightforward approach, combining short reads with reads of up to 30 kb length, resulted in the *ab initio* assembly of two contigs with a length of 116,666 and 46,221 bp, respectively. These contigs had overlapping ends and could therefore easily be merged into one genome assembly with a final length of 166,425 bp and a GC content of 35.5%. The overlap between the two contigs was confirmed by site-specific PCR and Sanger sequencing. The coverage of the Illumina reads was 124 (138,058 aligned reads), and that of the nanopore sequencing reads was 125 (11,248 aligned reads out of 94,061; average length of aligned reads = 1848). The length distribution of the aligned reads and a histogram of the coverage distribution are shown Supplementary File 1.

Phylogenetic analysis of the genome sequence of TePV-1 and the amino acid sequences of the putative DNA polymerase (highest amino acid sequence identity, 74.3%, to flamingopox virus, MF678796.1) and DNA topoisomerase (highest amino acid sequence identity, 76.0%, to fowlpox virus, NC_002188.1) revealed that TePV-1 is most closely related to members of the genus *Avipoxvirus* (Fig. 2).

**Fig. 2:**
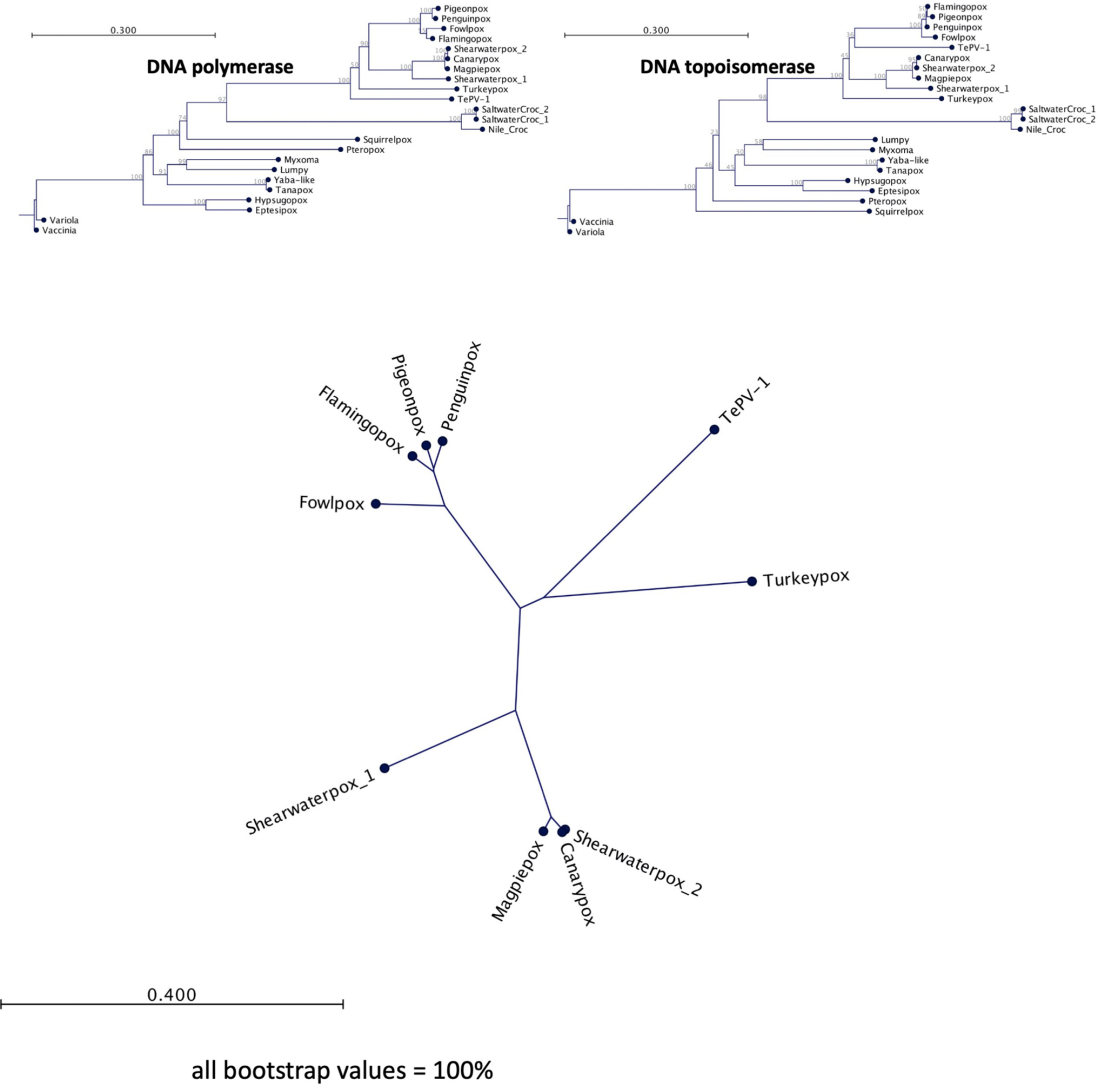
Phylogenetic relationship of TePV-1 to other members of the subfamily *Chordopoxvirinae*. (A) Neighbor-joining tree based on the DNA polymerase protein sequence. (B) Neighbor-joining tree based on the DNA topoisomerase protein sequence. (C) Neighbor-joining tree of the full genome sequences based on avipoxviruses. All branches had a bootstrap value of 100. All trees were generated after multiple sequence alignment in CLC Workbench with 1,000 replicates. The GenBank accession numbers for sequences employed in this analysis are as follows: canarypox virus, NC_005309.1; eptesipox virus, NC_035460.1; flamingopox virus, MF678796.1; fowlpox virus, NC_002188.1; hypsugopox virus, MK860688.1; lumpy skin disease virus, NC_003027.1; magpiepox virus, MK903864.1; myxoma virus, NC_001132.2; Nile crocodilepox virus, DQ356948.1; penguinpox virus, KJ859677.1; pigeonpox virus: KJ801920.1; pteropox virus, NC_030656.1; saltwater crocodile poxvirus 1, MG450915.1; saltwater crocodile poxvirus 2, MG450916.1; shearwaterpox virus 1, KX857216.1; shearwaterpox virus 2, KX857215.1; squirrelpox virus, NC_022563.1; tanapox virus, EF420157.1; turkeypox virus, KP728110.2; vaccinia virus, NC_006998.1; variola virus, NC_001611.1; Yaba-like disease virus, NC_002642.1.

Using CLC Workbench, 154 open reading frames coding for ≥100 amino acids were detected, and these sequences were compared to the proteome of other avipoxviruses, using BLASTp. The results of this analysis are presented in Table 1. Apart from the presence of ankyrin repeat proteins at the very beginning and the end of the coding region of the TePV-1 genome, the ORF arrangement was the same as in other avipoxviruses, even though the TePV-1 genome is significantly shorter. Interestingly, nine reading frames coding for proteins of 101-206 aa could not be related to any other sequences based on amino acid sequence identity or the presence of conserved domains. Seven ORFs were found to encode proteins that are not related to other poxvirus proteins but are related to proteins found in eukaryotes (see Supplementary Table 1) and could have been acquired by horizontal gene transfer [18]. These sequences, as well as the assembly site of the two contigs and the transitions to the inverted terminal repeats were confirmed by specific PCR and Sanger sequencing.

**Table 1:** Annotation of ORFs in the genome of TePV-1 encoding proteins larger than 100 aa. The start and end of ORFs located on the positive strand are shown in bold. The length of each ORF is given in base pairs. "Protein" indicates the highest-rated BLAST hit for this ORF, which is further specified by its accession number (GenBank no.), location in the genome (ref loc), the % amino acid sequence identity (% identity), the alignment length (ali), the e-value and the species. AC, *Anolis carolinensis*; CG, *Cricetulus griseus*; CNPV, canarypox virus; EA, *Equus asinus*; FePV2, pigeonpox virus; FGPV, flamingopox virus; FWPV, fowlpox virus; LA, *Lingula anatina*; MPPV, magpiepox virus; N., ORF number; PEPV, penguinpox virus; PM, *Protobothrops mucrosquamatus*; SWPV, shearwaterpox virus; TKPV, turkeypox virus

N	Start	End	Length	Protein	GenBank no.	ref loc	% identity	ali	e-value	Species
1	**843**	**1463**	621	SWPV1-308, conserved hypothetical protein	ARF02867.1	323041..323703	59.545	220	2.43E-87	SWPV-1
2	1841	3643	1803	Ankyrin repeat family protein	AUD40350.1	271364..273295	33.667	600	4.68E-93	FGPV
3	3583	4626	1044	Serpin family protein	AUD40336.1	256018..257064	34.582	347	2.93E-74	FGPV
4	4670	6163	1494	Ankyrin repeat protein	YP_009046459.1	264529..266415	41.04	519	2.61E-122	FeP2
5	6183	7934	1752	Ankyrin repeat protein	YP_009046459.1	264529..266415	42.484	612	1.26E-157	FeP2
6	7991	8368	378	EFc-like protein	ALA62542.1	185548..185937	34.711	121	2.27E-25	TKPV
7	8412	8969	558	Ankyrin repeat protein	YP_009046231.1	281304..281861	39.572	187	8.05E-45	PEPV
8	9035	9832	798	Ankyrin repeat protein	AYO89833.1	274191..275423	42.146	261	9.34E-66	FWPV
9	**9321**	**9668**	348	No similarity found						
10	9853	11604	1752	Ankyrin repeat protein	YP_009046027.1	33882..35669	46.259	588	6.97E-175	PEPV
11	11632	12234	603	Hypothetical protein	YP_009046028.1	35800..36411	55.051	198	5.22E-85	PEPV
12	**12608**	**13561**	954	G-protein-coupled receptor family protein	AUD40130.1	29544..30545	36.824	296	3.84E-67	FGPV
13	13541	15733	2193	alkaline phosphodiesterase	AUD40134.1	33068..35515	42.665	743	0	FGPV
14	15749	16861	1113	SWPV1-041, DNase II-like protein	ARF02656.1	47782..48978	41.842	380	2.09E-97	SWPV-1
15	16866	17279	414	Hypothetical protein	AUD40139.1	40555..40962	52.239	134	1.03E-48	FGPV
16	17303	17776	474	Hypothetical protein	ALA62391.1	22080..22559	43.312	157	4.06E-44	TKPV
17	17779	18213	435	dUTP pyrophosphatase	AUD40142.1	41945..42382	68.056	144	4.29E-72	FGPV
18	18219	18749	531	B-cell lymphoma 2	AUD40143.1	42434..42961	34.078	179	2.11E-28	FGPV
19	18785	19798	1014	Serpin family protein	YP_009046279.1	49969..50982	46.154	338	1.91E-111	FeP2
20	20154	21833	1680	DNA ligase	AYP74252.1	247565..249259	62.186	558	0	FWPV
21	21865	22923	1059	Putative serpin	AYP74251.1	246462..247523	50.992	353	1.06E-133	FWPV
22	22961	24649	1689	Semaphorin	YP_009046047.1	57639..59369	48.772	570	0	PEPV
23	**24724**	**25494**	771	GNS1/SUR4	AUD40150.1	51121..51906	78.516	256	1.57E-157	FGPV
24	**25547**	**26011**	465	Late transcription factor VLTF-2	AXY04490.1	52429..52893	76.623	154	5.41E-89	FWPV
25	25648	25971	324	Rifampicin resistance protein	AXY04491.1	52647..52856	76.562	64	1.14E-27	FWPV
26	**26032**	**27693**	1662	Rifampicin resistance protein	ALA62402.1	33712..35373	75.769	553	0	TKPV
27	**27710**	**28579**	870	mRNA capping enzyme	YP_009046288.1	61089..61958	79.585	289	1.20E-174	FeP2
28	28576	29709	1134	Class I histocompatibility antigen, F10 alpha chain-like	XP_015684078.2		46	164	5.00E-20	PM
29	29667	30548	882	Major histocompatibility complex class I-related gene protein	XP_008122411.1		35	172	2.00E-16	CNPV
30	**30526**	**32436**	1911	NPH-1 transcription termination factor	AUD40154.1	55108..57021	78.74	635	0	FGPV
31	32409	33104	696	muT motif expression regulator	YP_009046053.1	65721..66398	62.667	225	3.39E-102	PEPV
32	33088	33723	636	mutT motif containing protein	AXY04496.1	58126..58821	71.226	212	3.25E-109	FWPV
33	34048	34533	486	RNA polymerase subunit RPO18	YP_009046293.1	66549..67034	83.23	161	8.20E-98	FeP2
34	34520	36421	1902	Early transcription factor small subunit	AUD40159.1	60145..62046	90.047	633	0	FGPV
35	36402	38759	2358	NTPase	AUD40160.1	62027..64402	79.267	791	0	FGPV
36	38788	39444	657	Uracil DNA glycosylase	AXY04504.1	67861..68517	76.389	216	6.82E-127	FWPV
37	39461	40600	1140	SWPV1-075, conserved hypothetical protein	ARF02682.1	81697..82893	41.071	392	1.85E-88	SWPV-1
38	**40859**	**41215**	357	Putative glutathione peroxidase	QGM48717.1	80344..80754	68.908	119	1.91E-59	MPPV
39	41169	41540	372	Hypothetical protein	NP_955112.1	100368..100847	44.898	98	4.02E-29	CNPV
40	41859	42653	795	Virion protein	YP_009046067.1	80684..81493	47.212	269	3.94E-82	PEPV
41	**42841**	**43218**	378	Glutaredoxin	ALA62425.1	53825..54202	68	125	9.15E-62	TKPV
42	43161	43859	699	Putative transcriptional elongation factor	AUD40182.1	80826..81503	64	225	8.93E-102	FGPV
43	**43853**	**44164**	312	Hypothetical protein	AXY04521.1	79590..79901	66.019	103	3.22E-48	FWPV
44	**44188**	**46041**	1854	SWPV1-097, putative metalloprotease	ARF02699.1	95995..97896	65.561	633	0	SWPV-1
45	46028	48064	2037	RNA helicase NPH-II	AYP74219.1	203411..205456	72.485	676	0	FWPV
46	**48079**	**49338**	1260	Virion core proteinase	AYP74218.1	202111..203376	75.059	421	0	FWPV
47	**49339**	**50508**	1170	DNA-binding protein	YP_009046080.1	94650..95822	63.333	390	0	PEPV
48	50376	50708	333	No similarity found						
49	**51076**	**51828**	753	DNA-binding phosphoprotein	ALA62437.1	65235..66116	50.883	283	3.47E-101	TKPV
50	**52033**	**52968**	936	DNA-binding virion protein	QGM48748.1	99500..100432	81.935	310	0	MPPV
51	**53112**	**55055**	1944	Hypothetical protein	AUD40195.1	92674..94644	48.558	659	0	FGPV
52	**54985**	**55380**	396	Virion core protein	QGM48751.1	102503..102898	51.145	131	2.17E-47	MPPV
53	**55688**	**58654**	2967	DNA polymerase	AUD40198.1	95291..98260	74.291	988	0	FGPV
54	58646	59452	807	Hypothetical protein	AUD40199.1	98252..99100	71.269	268	3.94E-141	FGPV
55	59454	61169	1716	Hypothetical protein	AUD40200.1	99093..100808	71.979	571	0	FGPV
56	61190	66739	5550	B22R family protein	YP_009046096.1	118822..124590	47.199	1928	0	PEPV
57	**64481**	**64939**	459	No similarity found						
58	**66827**	**67372**	546	RNA polymerase subunit	AYP74202.1	171000..171548	79.006	181	1.84E-105	FWPV
59	**67375**	**69501**	2127	Hypothetical protein	AUD40205.1	118803..120956	55.23	717	0	FGPV
60	**69473**	**70891**	1419	PolyA polymerase large subunit PAP-L	YP_009046336.1	126407..127825	79.025	472	0	FeP2
61	70885	71202	318	DNA binding virion core phosphoprotein	AUD40207.1	122355..122699	70.175	114	9.14E-54	FGPV
62	**71281**	**71880**	600	Hypothetical protein	AYO89698.1	121669..122301	38.756	209	3.96E-46	FWPV
63	**71929**	**72375**	447	Hypothetical protein	YP_009046339.1	128991..129440	81.081	148	1.22E-88	FeP2
64	**72732**	**73865**	1134	SWPV1-124, putative palmitylated EEV envelope lipase	ARE67652.1	142768..143904	75.067	373	0	SWPV-1
65	**73897**	**75696**	1800	EEV maturation protein	AXY04551.1	129455..131347	46.825	630	0	FWPV
66	**75727**	**77052**	1326	Ser/Thr kinase	AYP74192.1	153725..155065	75.688	436	0	FWPV
67	**77030**	**77671**	642	Hypothetical protein	AXY04554.1	134129..134770	69.484	213	6.53E-106	FWPV
68	**78116**	**78655**	540	HAL3 domain containing protein	AXY04556.1	135430..135981	68.306	183	6.98E-96	FWPV
69	79127	79822	696	25-hydroxyvitamin D-1 alpha hydroxylase, mitochondrial	XP_003216736.1		38	121	1.00E-07	AC
70	79860	80702	843	Glutamate-rich protein 3 isoform X3	XP_013402139.1		37	101	6.00E-06	LA
71	**80169**	**80498**	330	No similarity found						
72	**80787**	**82091**	1305	Hypothetical protein	NP_955168.1	170735..172057	60.277	433	0	CNPV
73	**82286**	**82864**	579	SWPV1-137, conserved hypothetical protein	ARE67661.1	158056..158622	71.123	187	1.15E-98	SWPV-1
74	82817	83839	1023	Putative virion core protein	NP_955171.1	172777..173823	77.011	348	0	CNPV
75	**83894**	**84676**	783	Late transcription factor VLTF-1	AYP74173.1	129528..130310	87.308	260	3.61E-177	FWPV
76	**84681**	**85688**	1008	Poxvirus myristoylprotein	AXY04569.1	160397..161407	60.417	336	1.45E-159	FWPV
77	**85689**	**86420**	732	SWPV2-ORF161, putative myristylated IMV envelope protein	ARE67397.1	201657..202388	86.364	242	4.06E-165	SWPV-2
78	86699	87607	909	Hypothetical protein	YP_009046124.1	171203..172108	75.908	303	0	PEPV
79	**87633**	**88391**	759	DNA-binding virion core VP8	YP_009046362.1	156784..157545	67.984	253	1.64E-132	FeP2
80	**88392**	**88772**	381	Hypothetical protein	AYO89725.1	164799..165188	62.016	129	1.32E-54	FWPV
81	**88729**	**89169**	441	Putative IMV membrane protein	QGM48809.1	169617..170063	76.224	143	4.18E-80	MPPV
82	**89186**	**90079**	894	PolyA polymerase small subunit	AUD40238.1	158947..159873	70.27	296	4.29E-158	FGPV
83	**90076**	**90636**	561	RNA polymerase subunit 22	AUD40239.1	159870..160430	75.275	182	3.22E-102	FGPV
84	90629	91039	411	Membrane protein	AXY04578.1	166439..166852	77.686	121	4.38E-70	FWPV
85	**91075**	**94941**	3867	RNA polymerase subunit RPO147	YP_009046131.1	175641..179504	88.276	1288	0	PEPV
86	94945	95445	501	Protein tyrosine phosphatase	YP_009046369.1	164161..164661	79.518	166	1.86E-100	FeP2
87	**95459**	**96028**	570	Putative viral membrane protein	AXY04581.1	171281..171853	79.894	189	1.78E-117	FWPV
88	96025	96891	867	Virion envelope protein (p35)	YP_009046371.1	165428..166429	50.617	324	1.64E-121	FeP2
89	96892	99279	2388	RNA polymerase-associated protein 94	AUD40245.1	166990..169389	79.099	799	0	FGPV
90	**99382**	**99870**	489	VLTF-4	YP_009046373.1	168979..169503	46.286	175	3.66E-34	FeP2
91	**99871**	**100821**	951	SWPV2-ORF177, DNA topoisomerase	ARE67414.1	217385..218335	75.633	316	0	SWPV-2
92	**100826**	**101287**	462	SWPV1-166, conserved hypothetical protein	ARE67699.1	196050..196511	58.17	153	5.47E-61	SWPV-1
93	**101533**	**104082**	2550	mRNA capping enzyme large subunit	AXY04588.1	177778..180333	75.972	849	0	FWPV
94	104052	104474	423	CNPV194 virion protein	NP_955217.1	228919..229341	56.835	139	4.89E-50	CNPV
95	**104492**	**105904**	1413	Sodium-dependent lysophosphatidylcholine symporter 1 isoform X2	XP_027254970.1		47	473	2.00E-135	CG
96	**106631**	**107491**	861	p28-like protein	AYP74141.1	99505..100464	51.736	288	5.16E-114	FWPV
97	**107493**	**108848**	1356	CNPV206 putative photolyase	NP_955229.1	237537..238955	65.778	450	0	CNPV
98	**108875**	**109471**	597	N1R/p28 family protein	YP_009046151.1	198797..199537	33.166	199	2.99E-29	PEPV
99	**109519**	**110163**	645	CNPV209 N1R/p28-like protein	NP_955232.1	240422..241354	33.005	203	9.90E-36	CNPV
100	**110429**	**110941**	513	Hypothetical protein	ALA62492.1	122400..122924	34.969	163	6.26E-24	TKPV
101	**110916**	**112124**	1209	SWPV1-208, C4L/C10L-like protein	ARF02782.1	232534..233796	43.796	411	1.05E-115	SWPV-1
102	110927	111544	618	No similarity found						
103	112126	112803	678	Late transcription factor VLTF-3	AXY04607.1	198622..199299	88.889	225	2.66E-149	FWPV
104	113020	114966	1947	Virion core protein P4b	AXY04609.1	199529..201502	80.864	648	0	FWPV
105	115027	115521	495	Immunodominant virion protein	ALA62497.1	130871..131458	34.01	197	6.65E-30	TKPV
106	**115560**	**116054**	495	RNA polymerase subunit RPO19	YP_009046400.1	196511..197020	76.923	169	1.69E-89	FeP2
107	116049	117170	1122	SWPV2-ORF229, conserved hypothetical protein	ARE67480.1	268134..269255	65.952	373	2.06E-179	SWPV-2
108	**116797**	**117099**	303	No similarity found						
109	117173	119299	2127	Early transcription factor large subunit VETF-L	AYO90282.1	203339..205468	83.216	709	0	FWPV
110	**118121**	**118438**	318	No similarity found						
111	**119354**	**120256**	903	Intermediate transcription factor 3	AUD40279.1	203735..204640	79.07	301	0	FGPV
112	120449	123115	2667	Virion core protein P4a	AXY04616.1	207389..210064	73.46	893	0	FWPV
113	**123132**	**123908**	777	Hypothetical protein	AXY04617.1	210082..210906	74.088	274	3.08E-142	FWPV
114	123903	124352	450	Virion protein	QGM48882.1	229297..229803	56.875	160	7.46E-54	MPPV
115	125401	126492	1092	Putative myristoylated membrane protein	AUD40289.1	210114..211220	69.919	369	0	FGPV
116	126500	127078	579	Phosphorylated virion membrane protein	AXY04624.1	213785..214381	72.727	198	2.15E-100	FWPV
117	**127096**	**128472**	1377	CNPV257 DNA helicase, transcriptional elongation	NP_955280.1	286822..288210	74.891	458	0	CNPV
118	128696	129028	333	Conserved hypothetical protein	QGM48893.1	234410..234748	82.143	112	1.09E-62	MPPV
119	**129027**	**130310**	1284	Processivity factor	YP_009046183.1	227993..229291	59.767	430	0	PEPV
120	**130313**	**130765**	453	Holliday junction resolvase	AUD40295.1	215103..215588	67.105	152	1.83E-75	FGPV
121	**130785**	**131936**	1152	Intermediate transcription factor VITF-3	QGM48896.1	236523..237674	74.413	383	0	MPPV
122	**131963**	**135436**	3474	RNA polymerase subunit RPO132	YP_009046420.1	213357..216830	90.147	1157	0	FeP2
123	135425	136993	1569	A-type inclusion like protein	NP_955287.1	295207..297018	55.017	598	0	CNPV
124	**135968**	**136303**	336	No similarity found						
125	137029	138375	1347	A-type inclusion protein	YP_009046188.1	236280..237698	49.895	477	4.20E-170	PEPV
126	138376	138798	423	Hypothetical protein	AYP74103.1	57016..57438	72.857	140	2.63E-78	FWPV
127	138802	139713	912	RNA polymerase subunit RP035	AXY04635.1	226511..227419	60.726	303	2.60E-138	FWPV
128	**140010**	**140351**	342	Hypothetical protein	YP_009046427.1	221822..222163	61.947	113	6.59E-48	FeP2
129	**140352**	**140714**	363	Hypothetical protein	AYP74099.1	55034..55393	45.763	118	1.49E-35	FWPV
130	140703	141446	744	Virion assembly protein	YP_009046195.1	240113..241027	84.836	244	6.67E-161	PEPV
131	**141490**	**142044**	555	SWPV1-245, C-type lectin-like EEV protein	ARF02806.1	267315..267863	61.932	176	4.63E-81	SWPV-1
132	**142089**	**142520**	432	Hypothetical protein	NP_955296.1	302592..303416	38.095	147	4.07E-24	CNPV
133	**142505**	**143281**	777	Hypothetical protein	AXY04907.1	232786..233643	48	250	1.63E-75	FWPV
134	143273	143875	603	Hypothetical protein	AYP74093.1	49333..50022	37.864	206	1.42E-35	FWPV
135	**143925**	**145406**	1482	Prostacyclin synthase isoform X1	XP_008108335.1		45	486	1.00E-128	AC
136	**145447**	**145944**	498	SWPV2-ORF270, putative interleukin binding protein	ARE67521.1	302929..303510	35.897	117	7.90E-11	SWPV-2
137	**145928**	**146287**	360	Epidermal growth factor like protein	YP_009046207.1	251214..251585	40	100	3.24E-22	PEPV
138	**146287**	**147168**	882	Ser/Thr protein kinase	ALA62528.1	164710..165612	59.932	292	7.44E-135	TKPV
139	**147451**	**148422**	972	Hypothetical protein	AXY04659.1	243703..244689	48.287	321	1.65E-102	FWPV
140	**148454**	**149758**	1305	Ankyrin repeat protein	YP_009046446.1	240328..241650	40.139	431	2.75E-99	FeP2
141	**149820**	**152030**	2211	Ankyrin repeat protein	AXY05185.1	248089..250332	41.384	737	0	FWPV
142	**152033**	**152608**	576	Ankyrin repeat protein	YP_009046450.1	247134..248219	48.408	157	7.36E-51	FeP2
143	**152568**	**153041**	474	Zinc finger protein 709-like isoform X3	XP_014701435.1		48	119	9.00E-19	EA
144	**153057**	**154364**	1308	SWPV1-279, ankyrin repeat protein	ARF02838.1	294643..295941	39.25	400	4.78E-96	SWPV-1
145	**154387**	**155430**	1044	Serpin family protein	AUD40336.1	256018..257064	39.093	353	2.95E-80	FGPV
146	**155482**	**156876**	1395	Ankyrin repeat family protein	AUD40338.1	257748..259259	50	462	2.60E-153	FGPV
147	156861	157838	978	CNPV039 G protein-coupled receptor-like protein	NP_955062.1	46721..47704	40.122	329	2.49E-85	CNPV
148	157330	157659	330	No similarity found						
149	**158212**	**159471**	1260	Ankyrin repeat family protein	ALA62540.1	178533..179828	33.412	422	1.17E-67	TKPV
150	**159473**	**159784**	312	Late transcription factor VLTF-1	ALA62543.1	186082..186339	31.325	83	4.97E-09	TKPV
151	**159825**	**161417**	1593	Ankyrin repeat protein	YP_009046233.1	282988..284919	38.214	560	9.22E-117	PEPV
152	**161398**	**163155**	1758	SWPV2-ORF300, ankyrin repeat protein	ARE67552.1	332667..334556	38.548	620	1.06E-126	SWPV-2
153	**163142**	**164908**	1767	SWPV2-ORF300, ankyrin repeat protein	ARE67552.1	332667..334556	41.977	617	2.20E-157	SWPV-2
154	165195	165815	621	SWPV1-308, conserved hypothetical protein	ARF02867.1	323041..323703	59.545	220	2.43E-87	SWPV-1

Poxvirus-like lesions and infections have been described in various reptiles, including crocodilians, tortoises, chameleons, and tegus [6–11, 19]. Despite their description in the literature, they have not yet been characterized at the genetic level, except for poxviruses in Nile and saltwater crocodiles (Nile crocodilepox virus [CRV] and saltwater crocodilepox virus subtypes 1 and 2 [SwCRV1/2], respectively) [10, 11]. This first report of the genome sequence of a poxvirus causing disease in a lizard reveals it to be most closely related to avipoxviruses. This is surprising, considering the phylogenetic distance between avian and reptilian species and the differences in homeostasis. The GC content of TePV-1 (35%) is also more similar to that of avipoxviruses than to the known crocodile-infecting poxviruses (62% for CRV, Sw-CRV-1 and -2) [10, 11]. Interestingly, the initial diagnostic PCR only was positive when a primer set for high-GC-content poxviruses was employed. This might indicate that poxviruses of Reptilia are quite variable. Therefore, additional research is warranted to examine the diversity of poxviruses of Reptilia and their species specificity and zoonotic potential.

## Supplementary Information

Below is the link to the electronic supplementary material.

Supplementary file1 Coverage of the assembled genome sequence for the short- and long-read next-generation sequencing approaches. The read length distribution for the long-read sequencing approach is shown (DOCX 14 KB)

Supplementary file2 ORFs encoding proteins related to eukaryotic proteins (JPG 485 KB)
