# Supplementary material for: Discovery of a phylogenetically distinct poxvirus in diseased Crocodilurus amazonicus (family Teiidae)
Source: Arch Virol. 2021 Feb 12;166(4):1183–91. doi: 10.1007/s00705-021-04975-6 (PMC7952365; doi:10.1007/s00705-021-04975-6)
Supplement: Supplementary file 1 — Supplementary file1 Coverage of the assembled genome sequence for the short- and long-read next-generation sequencing approaches. The read length distribution for the long-read sequencing approach is shown (DOCX 14 KB) [file 705_2021_4975_MOESM1_ESM.docx]

Additional file 2:

ORFs encoding proteins closest related to eukaryotic proteins.

| ORF | protein | | species | accession | % identity |
| --- | --- | --- | --- | --- | --- |
| 28 | class I histocompatibility antigen, F10 alpha chain-like | | *Protobothrops mucrosquamatus* | XP_015684078.2 | 46 |
| 29 | major histocompatibility complex class I-related gene protein | | *Anolis carolinensis* | XP_008122411.1 | 35 |
| 69 | 25-hydroxyvitamin D-1 alpha hydroxylase, mitochondrial | | *Anolis carolinensis* | XP_003216736.1 | 38 |
| 70 | glutamate-rich protein 3 isoform X3 | | *Lingula anatina* | XP_013402139.1 | 37 |
| 95 | sodium-dependent lysophosphatidylcholine symporter 1 isoform X2 | | *Cricetulus griseus* | XP_027254970.1 | 47 |
| 135 | prostacyclin synthase isoform X1 | | *Anolis carolinensis* | XP_008108335.1 | 45 |
| 143 | zinc finger protein 709-like isoform X3 | | *Equus asinus* | XP_014701435.1 | 48 |
|  | |  | | | |
