# Supplementary figures and images for: Discovery of a phylogenetically distinct poxvirus in diseased Crocodilurus amazonicus (family Teiidae)
Source: Arch Virol. 2021 Feb 12;166(4):1183–91. doi: 10.1007/s00705-021-04975-6 (PMC7952365; doi:10.1007/s00705-021-04975-6)

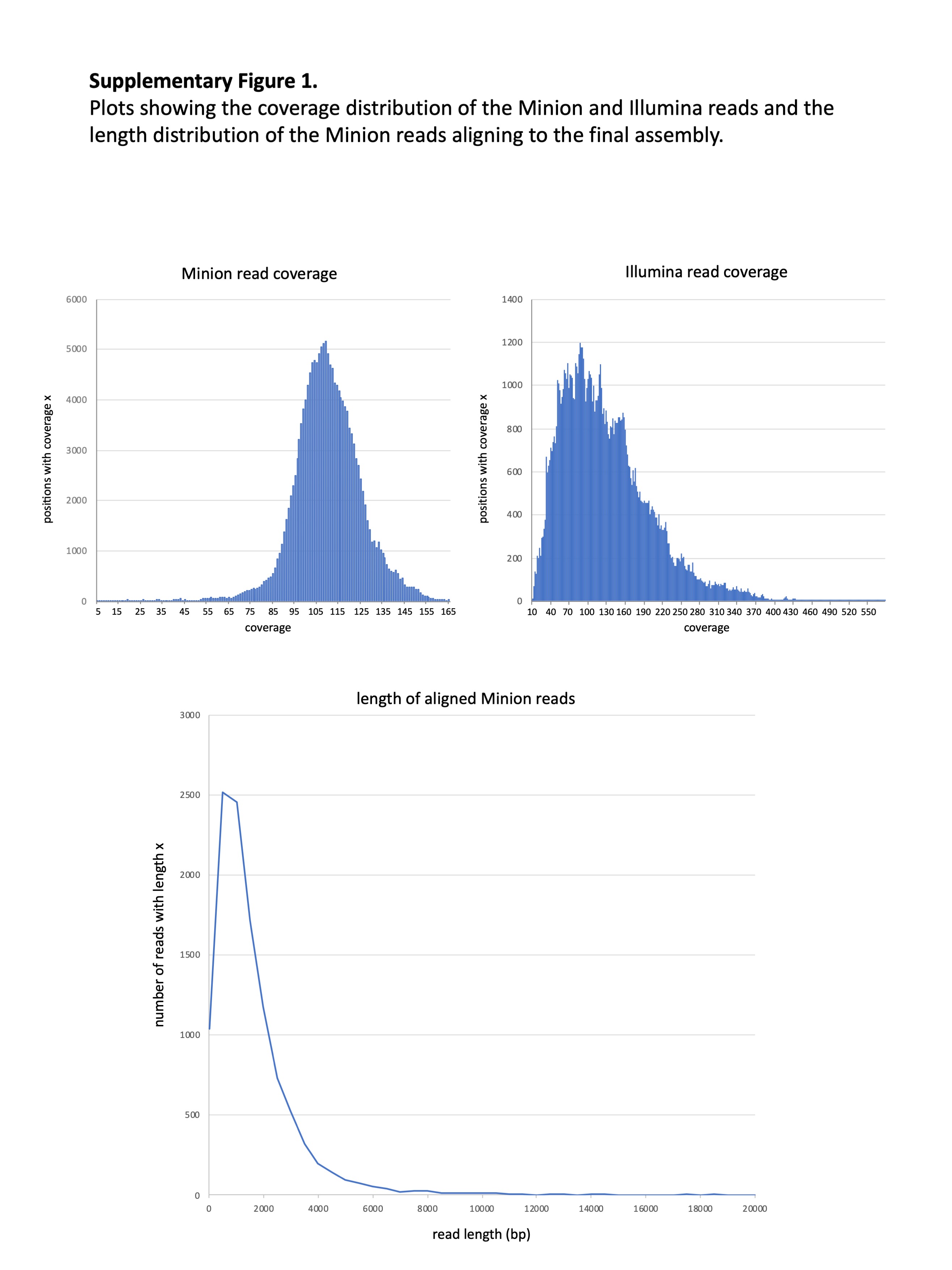

Supplement: Supplementary file 2 — Supplementary file2 ORFs encoding proteins related to eukaryotic proteins (JPG 485 KB) [file 705_2021_4975_MOESM2_ESM.jpg]
